# Discovery of a Novel Trifluoromethyl Diazirine Inhibitor of SARS-CoV-2 M^pro^

**DOI:** 10.3390/molecules28020514

**Published:** 2023-01-04

**Authors:** Andrea Citarella, Davide Moi, Martina Pedrini, Helena Pérez-Peña, Stefano Pieraccini, Claudio Stagno, Nicola Micale, Tanja Schirmeister, Giulia Sibille, Giorgio Gribaudo, Alessandra Silvani, Daniele Passarella, Clelia Giannini

**Affiliations:** 1 Department of Chemistry, University of Milan, Via Golgi 19, 20133 Milano, Italy; 2 Dipartimento di Scienze Chimiche e Geologiche, University of Cagliari, Cittadella Universitaria—S.S. 554 bivio per Sestu, 09042 Monserrato, Italy; 3 Department of Chemical, Biological, Pharmaceutical and Environmental Sciences, University of Messina, Viale Ferdinando Stagno D’Alcontres 31, 98166 Messina, Italy; 4 Department of Medicinal Chemistry, Institute of Pharmaceutical and Biomedical Sciences, Johannes Gutenberg University, Staudinger Weg 5, 55128 Mainz, Germany; 5 Department of Life Sciences and Systems Biology, University of Turin, Via Accademia Albertina 13, 10123 Torino, Italy

**Keywords:** SARS-CoV-2 M^pro^, diazirines, coronavirus, SARS-CoV-2, COVID-19, cysteine proteases, enzymatic inhibitors

## Abstract

SARS-CoV-2 M^pro^ is a chymotrypsin-like cysteine protease playing a relevant role during the replication and infectivity of SARS-CoV-2, the coronavirus responsible for COVID-19. The binding site of M^pro^ is characterized by the presence of a catalytic Cys145 which carries out the hydrolytic activity of the enzyme. As a consequence, several M^pro^ inhibitors have been proposed to date in order to fight the COVID-19 pandemic. In our work, we designed, synthesized and biologically evaluated **MPD112**, a novel inhibitor of SARS-CoV-2 M^pro^ bearing a trifluoromethyl diazirine moiety. **MPD112** displayed in vitro inhibition activity against SARS-CoV-2 M^pro^ at a low micromolar level (IC_50_ = 4.1 μM) in a FRET-based assay. Moreover, an inhibition assay against PL^pro^ revealed lack of inhibition, assuring the selectivity of the compound for the M^pro^. Furthermore, the target compound **MPD112** was docked within the binding site of the enzyme to predict the established intermolecular interactions in silico. **MPD112** was subsequently tested on the HCT-8 cell line to evaluate its effect on human cells’ viability, displaying good tolerability, demonstrating the promising biological compatibility and activity of a trifluoromethyl diazirine moiety in the design and development of SARS-CoV-2 M^pro^ binders.

## 1. Introduction

**Figure 1:**
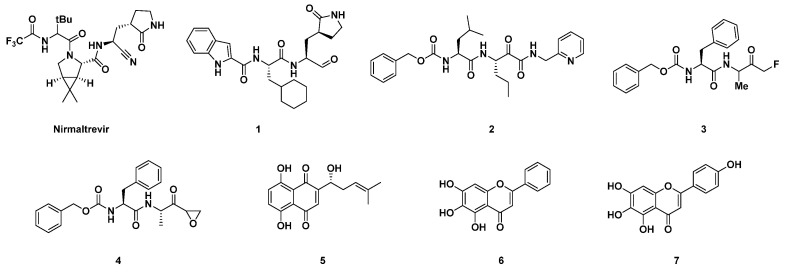
Examples of different chemical classes of SARS-CoV-2 M^pro^ inhibitors. A covalent mechanism of action was observed for M^pro^ inhibitors bearing electrophilic fragments, such as aldehydes (**1**), α-ketoamides (**2**), fluoromethyl ketones (**3**), and epoxyketones (**4**). A non-covalent inhibition of M^pro^ was reported for **5**, **6** and **7**.

The replication cycle of SARS-CoV-2 is sensitively correlated with the activity of its main protease (SARS-CoV-2 3CL^pro^/M^pro^), which nowadays represents an appealing target for the development of antiviral drugs for the treatment of severe COVID-19 [1,2]. M^pro^, together with the other fundamental SARS-CoV-2 protease, PL^pro^ [3], accomplishes its proteolytic activity towards the 1a/1ab polyprotein (pp) by hydrolyzing it into 16 mature non-structural proteins (NSPS) [4]. The latter are involved in essential roles in the context of the early phases of the SARS-CoV-2 replication cycle, such as the synthesis of viral RNAs and the rearrangement of host cell cytoplasmic organelles to generate platforms conducive to viral genome replication and, additionally, the expression of structural proteins (i.e., envelope, membrane, spike, and nucleocapsid), and the assembly and release of new viral particles. The homodimer protease is organized into protomers A and B which, upon dimerization and activation, assume an appropriate conformation to correctly fulfill the catalytic function [5]. Cys145 and His41 of M^pro^ designate the catalytic dyad in which the thiol (-SH) group of Cys, after deprotonation by His, realizes the proteolytic mechanism to the substrate. The interruption of the catalytic activity of M^pro^ could be thus a valuable medicinal chemistry strategy for the development of anti-coronavirus direct-acting antiviral (DAA) drugs, such as Paxlovid, an association of the M^pro^ inhibitor nirmaltrevir (Figure 1) and ritonavir, for the treatment of acute forms of COVID-19 [6]. A conspicuous number of peptidomimetics and small-molecule inhibitors of M^pro^ have been discovered since 2020. More recently, also some noteworthy metal-based complexes have appeared in the panorama of possible agents for the treatment of COVID-19 [7,8]. Most of the covalent inhibitors reported in literature so far are peptidomimetics able to block the activity of M^pro^ by forming an adduct with its Cys145 residue (Figure 1). They possess an electrophilic functional group at the C-terminus, called a “warhead”, usually endowed with great affinity for the thiol group [9]. A covalent mechanism of action is thus observed for M^pro^ inhibitors bearing electrophilic fragments, such as aldehydes (**1**), α-ketoamides (**2**), fluoromethyl ketones (**3**) (FMKs), and epoxyketones (**4**), inter alia (Figure 1) [2]. On the other hand, several non-covalent inhibitors of SARS-CoV-2 M^pro^ have been reported in the literature as well, and some of them, i.e., **5**, **6** and **7**, are depicted in Figure 1. They all turned out to be effective in the in vitro enzymatic assay against M^pro^; in particular, **5** was able to bind the protease in the same catalytic pocket as for the covalent inhibitors, interacting with the Cys145 residue through H bonds (Figure 1) [10].

**Scheme 1:**
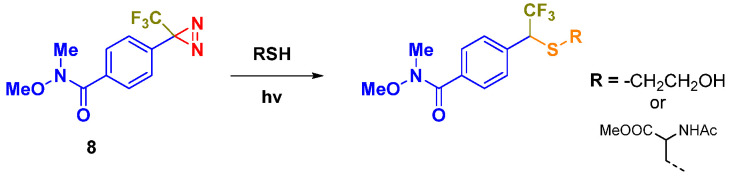
Examples of chemical reactivity mechanism of a diazirine moiety via photoactivation.

Diazirines are considered relevant chemical scaffolds usually employed as photo-labeling agents [11]. Their high stability—mainly the aryl-based diazirines—makes them appropriate for such a purpose, and to date several applications of diazirine-based compounds in organic synthesis and medicinal chemistry have been found [12,13]. West et al. described the reactivity of the trifluoromethyl diazirine scaffold with amino acid side chains, in order to evaluate the labeling preferences of diazirines for peptidic biomolecules [14]. They found out that the diazirine **8**, under photoactivation conditions, produces insertion products with all ordinary amino acids, via carbene formation, affording the highest yield by reacting with cysteine. The reaction occurs between the cysteine thiol group and the electrophilic diazirine carbon (Scheme 1—path 1).

In the present work, we discovered **MPD112**, a trifluoromethyl diazirine-based compound able to selectively inhibit the enzymatic activity of SARS-CoV-2 M^pro^ and endowed with a notable level of safety with regard to the viability of human cells. An incubation test with *N*-acetyl cysteine over 24 h revealed no addition of the thiol group to **MPD112**, confirming the stability of the diazirine moiety in the absence of photoactivation. In silico studies highlighted the most important interactions of **MPD112** within the SARS-CoV-2 M^pro^ binding pocket, supporting a plausible non-covalent inhibition mechanism.

## 2. Results and Discussion

**Figure 2:**
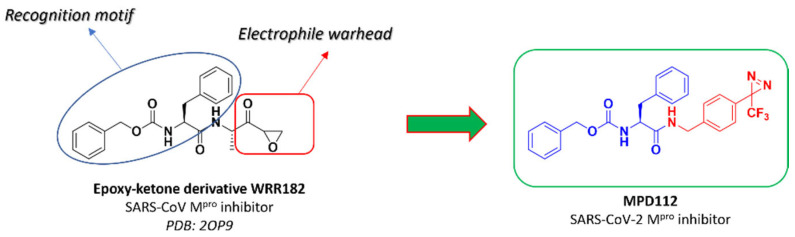
Design of the SARS-CoV-2 M^pro^ inhibitor **MPD112**.

The design of our inhibitor **MPD112** was accomplished starting from the analysis of the crystallographic structure of an epoxyketone inhibitor **WRR182** with SARS-CoV M^pro^ (Figure 2) [15]. The *N*-terminal carbobenzyloxy-phenylalanine residue has often been representative of several peptide-based inhibitors of SARS-CoV-2 M^pro^ described in the literature, especially α-fluorinated ketones and epoxyketones [16]. Therefore, we decided to maintain such functionality in the structure of the final compound and implemented the replacement of the epoxyketone moiety of the lead compound with the diazirine scaffold, connecting the latter to the steady fragment via an amide bond and a benzyl linker (Figure 2).

**Scheme 2:**
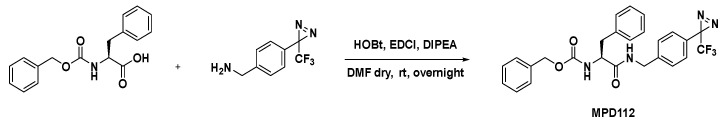
Synthesis of MPD112.

The synthesis of **MPD112** was carried out following an easy amide coupling between Z-Phe-OH and 4-[3-(trifluoromethyl)-3H-diazirin-3-yl]benzylamine hydrochloride, mediated by HOBt/EDCI/DIPEA in dry DMF, as reported in Scheme 2. Characterization of the compound by ^1^H, ^13^C NMR and HRMS confirmed the desired structure. Its purity (>95%) was assured by HPLC analysis. The diazirine building block was synthesized according to a previously reported procedure [17]. Copies of NMR and MS spectra, together with HPLC traces, are included in the Supplementary Materials.

**MPD112** underwent a preliminary screening at 20 μM for its ability to inhibit in vitro the recombinant SARS-CoV-2 M^pro^ activity. The preliminary assays showed promising full inhibition results. Then, continuous assays revealed that **MPD112** acts as a potent micromolar inhibitor (K_i_ = 2.3 ± 0.34; IC_50_ = 4.1 ± 0.6 μM). The same enzymatic evaluation of **MPD112** against the recombinant SARS-CoV-2 PL^pro^ revealed, in contrast, no inhibition, elucidating a selectivity for the main protease. Reactivity with the thiol group was investigated incubating **MPD112** (1 equiv) with N-acetyl cysteine (1.1 equiv) in a buffer (pH = 7.4) containing 10% acetonitrile at 37 °C, and the results were analyzed using LC-MS. No addition of cysteine was observed after 24 h, confirming the stability of the diazirine moiety, leading us to support a non-covalent mechanism of action [18].

**Figure 3:**
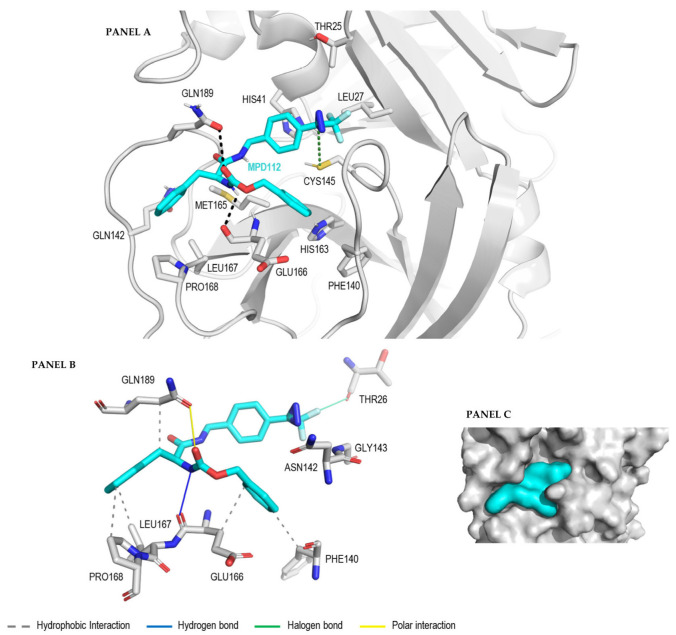
Best binding mode of **MPD112** obtained by Autodock Vina when bound to the active site of the M^pro^ of SARS-CoV-2 (PDB: 7NG3) shown in sticks mode (panel **A**), the protein–ligand interaction profile of the complex (panel **B**) and its representation in surface mode (panel **C**).

The binding modes of **MPD112** when in complex with M^pro^ were explored with a molecular docking approach. Therefore, we performed docking experiments in which the results show the best predicted binding mode of **MPD112** when bound to M^pro^ (PDB: 7NG3) using Autodock Vina as the docking software and Chimera as the docking interface. In Autodock Vina, the docking score represents a binding free energy prediction and allows us to rank the binding modes acquired by the same molecule when bound to a certain binding site. The results of the best **MPD112** conformer when tested in the binding site of M^pro^ presents a negative docking score (−7.8 kcal/mol). Then, further qualitative evaluation was carried out to understand the nature of this predicted score. The analysis of the resulting binding mode of **MPD112** when in complex with M^pro^ positions the trifluoromethyl diarizine moiety of **MPD112** in close proximity to the thiol group of Cys145 (Figure 3, panel A). This could be an explanation for its potent inhibitory activity. A more detailed investigation of the interactions established between **MPD112** and the residues contained in the binding site of the M^pro^ was then performed. First, the amino acid composition of the binding site was investigated. This site is composed of a mixture of hydrophobic and hydrophilic amino acids, where residues able to form hydrophobic interactions surround **MPD112**. This is important because in protein–ligand complexes, hydrophobic interactions can play a crucial role in the binding of the ligand to the protein. **MPD112** contains hydrophobic moieties, such as three aromatic groups, that can form hydrophobic interactions with the residues on the protein’s binding site and favor the protein–ligand binding. More specifically, we can observe in Figure 3 (panel B) that the benzyl carbamate group present in **MPD112** establishes hydrophobic interactions with the non-polar atoms of the side chain of Phe140 and Glu166. Moreover, the benzyl residue of **MPD112** forms hydrophobic interactions with the hydrophobic cyclic side chain of Pro168, the non-polar amino acid Leu167 and the non-polar region of the residue Gln189. Furthermore, the benzyl carbamate group present in **MPD112** establishes a hydrogen bond with the backbone oxygen atom of the residue Glu166. This hydrogen bond is one of the strongest interactions present in this protein–ligand complex, together with the halogen bond formed between the diazirine-substituted benzylamide group of compound **MPD112** and the side chain of Thr26. These two interactions permit the ligand to be more fixed to the M^pro^ site in the shown binding mode (Figure 3, panel B). Polar interactions as a general term to describe attractive or repulsive forces between the protein and the ligand that have an asymmetrical distribution of charge are also present in the M^pro^-**MPD112** complex. Moreover, a clear complementarity between the shape of **MPD112** and the binding site of M^pro^ was observed (Figure 3, panel C).

As a first step towards verifying the applicative potential of **MPD112**, its effect on human cell viability was analyzed by assessing the cytotoxic concentration (CC_50_) in HCT-8 cells exposed for 72 h to increasing concentrations of the compound. The determined CC_50_ was >400 μM, thus suggesting a notable level of safety as an added value of **MPD112**′s biological properties.

## 3. Experimental Section

### 3.1. General Experimental Information

Unless otherwise stated, reagents and solvents were purchased from Merck (Milan, Italy), Fluorochem (Hadfield, United Kingdom) or TCI (Zwijndrecht, Belgium) and used without further purification. All reactions were carried out in oven-dried glassware and with dry solvents, under nitrogen atmosphere, and were monitored by TLC on silica gel (Merck precoated 60F_254_ plates), with detection by UV light (254 nm) or by permanganate, or by HPLC. HPLC was performed on Agilent 1100 Series System using a Gemini 5 μM C18 110 Å LC Column 150 × 3 mm and a gradient of H_2_O/ACN from 5% ACN to 100% ACN in 40 min, flux of 1.0 mL/min and sample injection of 20 μL. The wavelength used for detection was 220 nm. Products were purified by flash column chromatography, using silica gel Merck 60 (230–400 mesh) as the stationary phase. ^1^H NMR and ^13^C NMR spectra were recorded on a Bruker Avance Spectrometer (400 MHz), using commercially available deuterated (CD_2_Cl_2,_ Chloroform-*d*, DMSO-*d*_6_) solvent at room temperature. Chemical shifts are reported in parts per million (δ ppm), compared to TMS as an internal standard. Multiplicities in ^1^H NMR are reported as follows: s—singlet, d—doublet, t—triplet, m—multiplet, br—broad. Data for ^13^C NMR are reported in chemical shift (δ ppm). High-resolution mass spectra (HRMS) were recorded using the Q-ToF Synapt G2-Si HDMS Acquity UPLC I-Class Photodiode Detector Array (PDA) (Waters).

### 3.2. Synthesis of MPD112

Benzyl (S)-(1-oxo-3-phenyl-1-((4-(3-(trifluoromethyl)-3H-diazirin-3-yl)benzyl)amino)propan-2-yl)carbamate (MPD112), Z-Phe-OH (66 mg, 0.22 mmol, 1.1 equiv) and HOBt (30 mg, 0.22 mmol, 1.1 equiv) were dissolved in dry DMF (1.0 mL) and DIPEA (0.12 mL, 0.7 mmol, 3.5 equiv) was added. After 30 min, EDCI (43 mg, 0.22 mmol, 1.1 equiv) and 4-[3-(trifluoromethyl)-3*H*-diazirin-3-yl]benzylamine hydrochloride (50 mg, 0.2 mmol, 1.0 equiv) were added and the mixture was left to stir at room temperature overnight. The reaction mixture was then diluted with ethyl acetate (5 mL) and was washed with saturated aqueous solution of NH_4_Cl (2 × 5 mL) and saturated aqueous solution of NaHCO_3_ (2 × 5 mL). The organic layer was dried with anhydrous Na_2_SO_4_, filtered and concentrated under reduced pressure. The crude substance was triturated with Et_2_O followed by filtration of the solid to afford MPD112 as a white amorphous solid (69% yield, 76 mg); ^1^H NMR (400 MHz, CD_2_Cl_2_): δ (ppm) 7.36–7.22 (m, 8H, Ar H), 7.17–7.09 (m, 6H, Ar H), 6.29 (bs, 1H, NH), 5.39 (bs, 1H, N*H*), 5.04 (s, 2H, OC*H*_2_), 4.43–4.27 (m, 3H, HNC*H*_2_ and α-C*H*), 3.06 (d, *J* = 7.0 Hz, 2H, PhC*H*_2_). ^13^C NMR (100 MHz, CD_2_Cl_2_): δ (ppm) 171.2, 140.5, 139.7, 136.9, 136.8, 129.7, 129.1, 128.9, 128.6, 128.3, 128.3, 127.4, 127.1, 124.0, 121.2, 67.4, 56.8, 43.1, 38.8. HPLC rt: 20.2 min. MS (ESI), *m/z* [M+H]^+^: 497.31. HRMS (ESI), *m/z* [M+Na]^+^: calculated for C_26_H_23_F_3_N_4_NaO_3_^+^: 519.1614; found 519.1620.

### 3.3. Enzymatic Assay

The inhibitory activity of the compounds was evaluated by means of a Förster resonance energy transfer (FRET)-based enzymatic cleavage assay on a TECAN Infinite F2000 PRO plate reader (Agilent Technologies, Santa Clara, USA) using white flat-bottom 96-well microtiter plates (Greiner bio-one, Kremsmünster, Austria). Recombinant SARS-CoV-2 Mpro was expressed and purified as previously described [19], whereas the peptidic substrate Dabcyl-KTSAVLQ↓SGFRKME-Edans (TFA salt) was obtained from a commercial source (Genescript, New Jersey, USA). The arrow indicates the cleavage position. The proteolytic activity of the SARS-CoV-2 M^pro^ was measured by monitoring the increasing fluorescence of SGFRKME-Edans upon hydrolytic shedding of the quencher Dabcyl-KTSAVLQ at 25 °C with a 335 nm excitation filter and a 493 nm emission filter. Each well contained 200 µL composed of 185 µL of reaction buffer (20 mM Tris pH 7.5, 0.1 mM EDTA, 1 mM DTT and 200 mM NaCl), 5 µL of SARS-CoV-2 M^pro^ in enzyme buffer at a final concentration of 50 nM together with 5 µL of the fluorogenic substrate (final concentration 25 μM) and 10 µL of the compounds present at a final concentration of 20 μM (screening assay) or at variable concentrations (IC_50_ assay). Specifically, for **MPD112**: 0–0.5–1–2.5–5–10–20–40 μM. DMSO was used as a negative control. Inhibitors and the substrate were dissolved and diluted in DMSO, leading to a final DMSO concentration of 7.5% (v/v). The compounds and enzyme were incubated for 10 min at 25 °C prior to substrate addition. The product released from substrate hydrolysis was monitored in 30 s increments over a period of 10 min. The related *K*_M_ value was determined in a separate experiment (33 µM). IC_50_ value was calculated with GraFit (Version 6.0.12; Erithacus Software Limited, East Grinstead, West Sussex, UK) by fitting the relative enzymatic activities blotted against the respective inhibitor concentration to the four-parameter equation. The *K*_i_ value was calculated by means of the Cheng–Prusoff equation.

### 3.4. Docking

The crystal structure of the main protease (M^pro^) from SARS-CoV-2 used for the docking experiments was obtained from the Protein Data Bank (PDB) entry with ID 7NG3. The first random three-dimensional structure of MPD112 was generated using the program MarvinSketch from ChemAxon^1^. Docking input files for the target protein M^pro^ and the ligand structure were prepared with UCSF Chimera 1.14^2^. The calculations were performed using AutoDock Vina^3^, which was run in UCSF Chimera 1.14 for the analysis and visualization of the results. AutoDock Vina considered all the atoms of the target site included in a cubic grid box with a grid spacing of 0.375 Å and a grid size of 20 Å for the geometry search within the docking process. The origin of the grid was positioned at the center of the M^pro^ binding site in which Cys145 is located. Finally, the resulting docking models were classified by the value of binding-free energy ∆G0 (kcal/mol), and the best solution with the lowest energy was selected. The images were rendered using PyMol 2.5.4^4^.

### 3.5. Cells and Cytotoxicity Assay

The human colorectal carcinoma HCT-8 cell line (ATCC CCL-244) was obtained from the American Type Culture Collection (ATCC) and maintained in Dulbecco’s modified Eagle medium (DMEM, Euroclone) supplemented with 10% fetal bovine serum (FBS, Euroclone), 2 mM glutamine, 1 mM sodium pyruvate, 100 U/mL penicillin, and 100 μg/mL streptomycin sulfate (Euroclone).

For the cytotoxicity assay, HCT-8 cells were seeded in 96-well plates (20,000 cells/well) and, after 24 h, exposed to increasing concentrations of **MPD112** or vehicle (DMSO) as a control. After 72 h of incubation, the number of viable cells was determined using the CellTiter-Glo Luminescent assay (Promega, Madison, WI, USA), as previously described [20].

## 4. Conclusions

To sum up, in our work we have synthesized **MPD112**, a new derivative endowed with a trifluoromethyl diazirine moiety and a potent inhibitory activity in vitro against SARS-CoV-2 M^pro^. Its inhibitory effect against the recombinant enzymatic target was evaluated in a FRET-based assay, showing micromolar values in terms of potency (IC_50_ = 4.1 ± 0.6 μM) and binding affinity (*K*i of 2.3 ± 0.34 μM). The molecule also showed great selectivity for SARS-CoV-2 M^pro^ in comparison to SARS-CoV-2 PL^pro^ and demonstrated a good level of safety in vitro against HTC-8 cells (CC_50_ > 400 μM). A docking analysis of the inhibitor inside the active cavity of M^pro^ showed an interesting complementarity, with the trifluoromethyl diazirine functionality in close contact with the thiol group of Cys145. This preliminary result represents a novel biological application of trifluoromethyl diazirine derivatives as SARS-CoV-2 M^pro^ inhibitors, and our efforts will be devoted to the continuous exploration of this functional group, with important applications in drug discovery.

## Data Availability

Data is contained within the article or Supplementary Material.

## Supplementary Materials

The following supporting information can be downloaded at: https://www.mdpi.com/article/10.3390/molecules28020514/s1, Copies of 1H NMR, 13C NMR and MS spectra, HPLC traces and the dose–response curve are included in the supplementary material.

## Sample Availability

Samples of the compound **MPD112** are available from the authors.
