# Supplementary material for: Discovery of a Novel Trifluoromethyl Diazirine Inhibitor of SARS-CoV-2 Mpro
Source: Molecules. 2023 Jan 4;28(2):514. doi: 10.3390/molecules28020514 (PMC9864213; doi:10.3390/molecules28020514)
Supplement: Supplementary file 1 [file molecules-28-00514-s001.zip › molecules-2093267-supplementary.pdf]

Supplementary materials for:

Discovery of a novel trifluoromethyl diazirine inhibitor of SARS-CoV-2 M^{pro}

Andrea Citarella,[1] Davide Moi,[2] Martina Pedrini,[1] Helena Pérez-Peña,[1] Stefano Pieraccini,[1] Claudio Stagno,[3] Nicola Micale,[3] Tanja Schirmeister,[4] Giulia Sibille,[5] Giorgio Gribaudo,[5] Alessandra Silvani,[1] Daniele Passarella[1] and Clelia Giannini,*[1]

[1] Department of Chemistry, University of Milan, Via Golgi 19, 20133, Milano, Italy

[2] Dipartimento di Scienze Chimiche e Geologiche, University of Cagliari, Cittadella Universitaria - S.S. 554 bivio per Sestu, Monserrato (CA), 09042, Italy

[3] Department of Chemical, Biological, Pharmaceutical and Environmental Sciences, University of Messina, Viale Ferdinando Stagno D'Alcontres 31, I-98166 Messina

[4] Department of Medicinal Chemistry, Institute of Pharmaceutical and Biomedical Sciences, Johannes Gutenberg University, Staudinger Weg 5, 55128 Mainz, Germany

[5] Department of Life Sciences and Systems Biology, University of Turin, Via Accademia Albertina 13, 10123, Torino, Italy

Correspondence: clelia.giannini@unimi.it

Table of contents

1. ^{1}H- and ^{13}C- NMR Spectra of MPD112 1
2. HPLC trace (Figure S1) and MS Spectra (Figure S2) of MPD112 2
3. Dose-response curve of MPD112 (Figure S3) 3

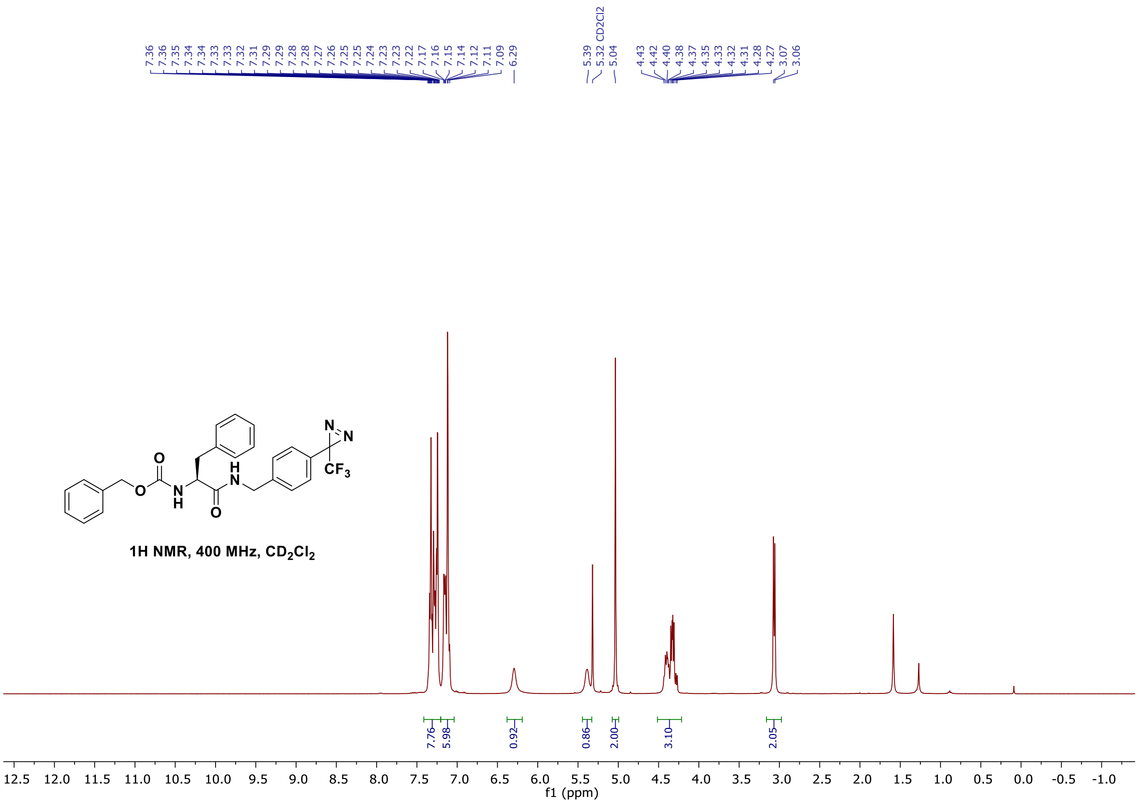
7.36 7.36 7.35 7.34 7.34 7.33 7.33 7.32 7.31 7.29 7.29 7.28 7.28 7.27 7.26 7.25 7.25 7.24 7.23 7.23 7.22 7.17 7.16 7.15 7.14 7.12 7.11 7.09 6.29
5.39 5.32 CD2Cl2 5.04
4.43 4.42 4.40 4.38 4.37 4.35 4.33 4.32 4.31 4.28 4.27 3.07 3.06
1H NMR, 400 MHz, CD_2Cl_2
7.76 5.98 0.92 0.86 2.00 3.10 2.05
f1 (ppm)

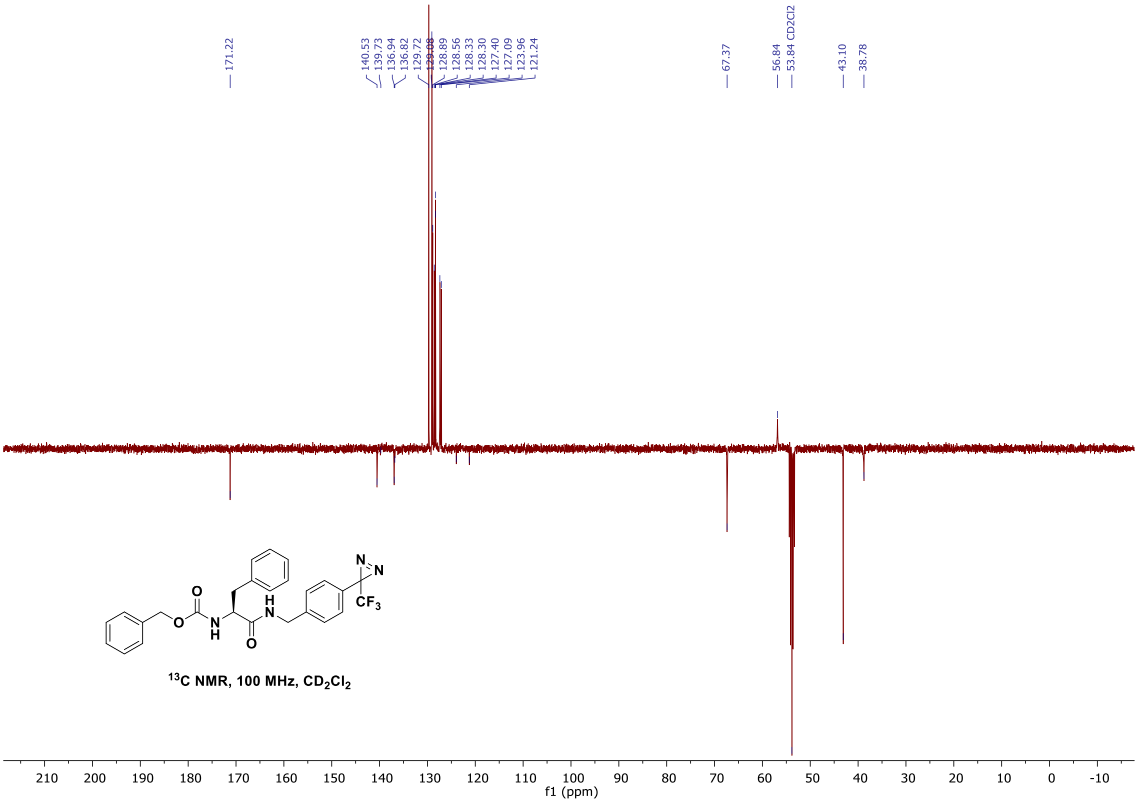
171.22
140.53 139.73 136.94 136.82 129.72 129.08 128.89 128.56 128.33 128.30 127.40 127.09 123.96 121.24
67.37
56.84 53.84 CD2Cl2
43.10 38.78
^{13}C NMR, 100 MHz, CD_2Cl_2
f1 (ppm)

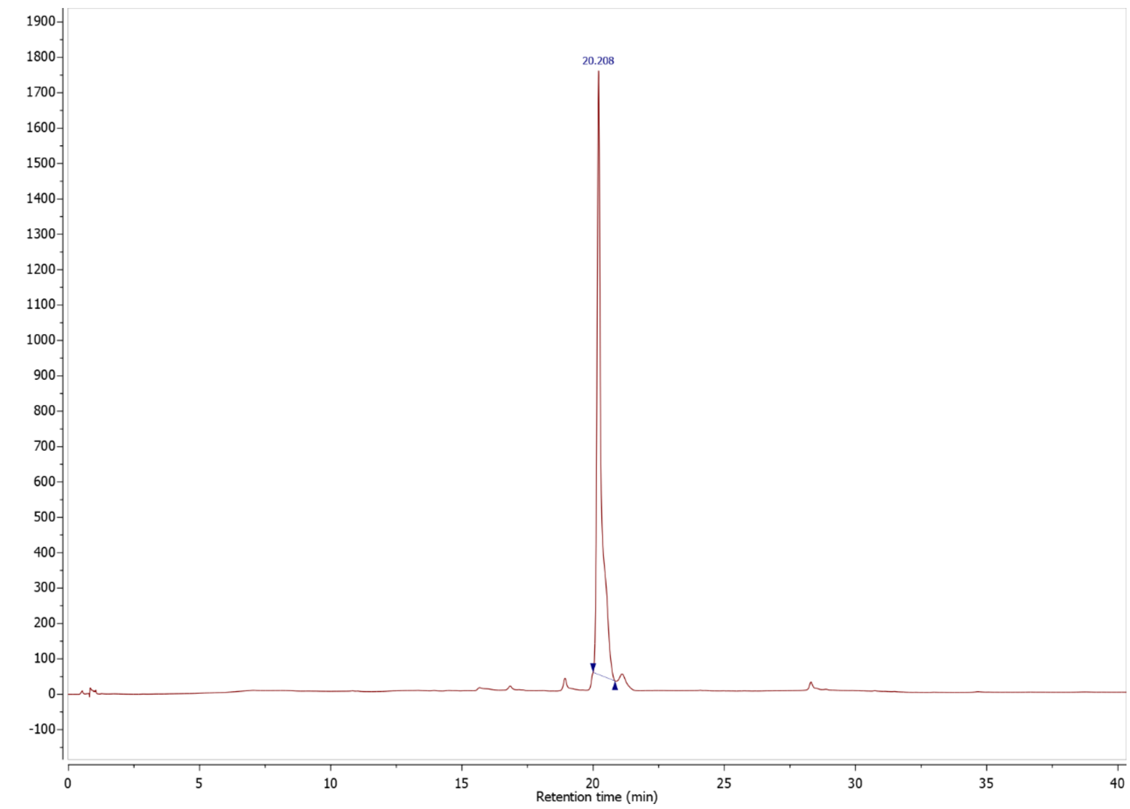

Figure S1. HPLC trace of MPD112.

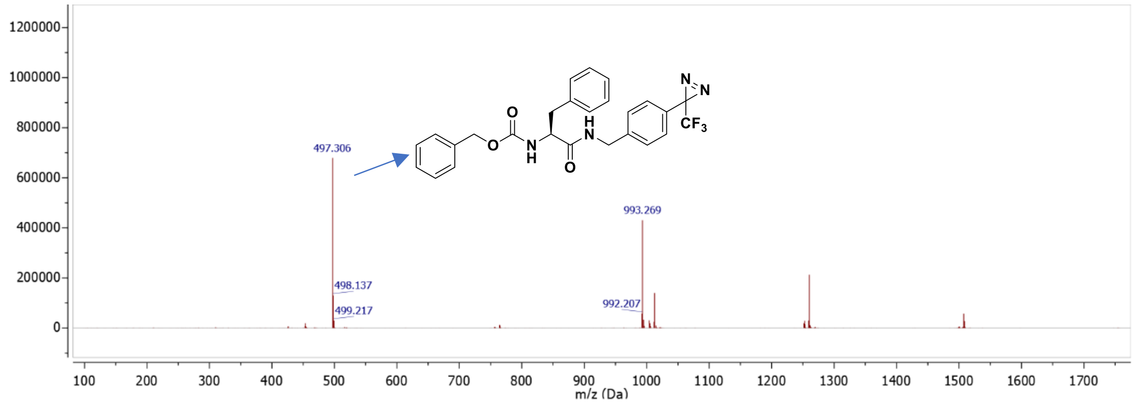

Figure S2. MS spectra of MPD112.

Predicted *m/z* $[M+H]^+$: 497.179, found 497.306.

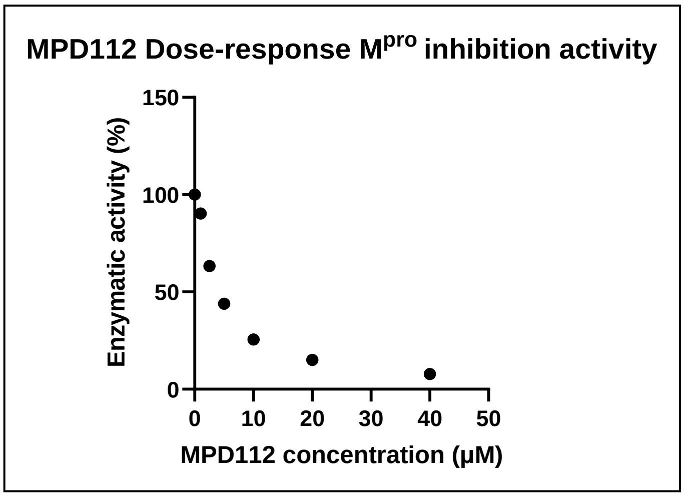

Figure S3. Dose-response curve of MPD112.
